# Role of Prenatal Hypoxia in Brain Development, Cognitive Functions, and Neurodegeneration

**DOI:** 10.3389/fnins.2018.00825

**Published:** 2018-11-19

**Authors:** Natalia N. Nalivaeva, Anthony J. Turner, Igor A. Zhuravin

**Affiliations:** ^1^I. M. Sechenov Institute of Evolutionary Physiology and Biochemistry, Russian Academy of Sciences, St. Petersburg, Russia; ^2^Faculty of Biological Sciences, School of Biomedical Sciences, University of Leeds, Leeds, United Kingdom; ^3^Research Centre, Saint-Petersburg State Pediatric Medical University, St. Petersburg, Russia

**Keywords:** prenatal hypoxia, learning, memory, brain plasticity, amyloid-degrading enzymes, neprilysin, Alzheimer's disease

## Abstract

This review focuses on the role of prenatal hypoxia in the development of brain functions in the postnatal period and subsequent increased risk of neurodegenerative disorders in later life. Accumulating evidence suggests that prenatal hypoxia in critical periods of brain formation results in significant changes in development of cognitive functions at various stages of postnatal life which correlate with morphological changes in brain structures involved in learning and memory. Prenatal hypoxia also leads to a decrease in brain adaptive potential and plasticity due to the disturbance in the process of formation of new contacts between cells and propagation of neuronal stimuli, especially in the cortex and hippocampus. On the other hand, prenatal hypoxia has a significant impact on expression and processing of a variety of genes involved in normal brain function and their epigenetic regulation. This results in changes in the patterns of mRNA and protein expression and their post-translational modifications, including protein misfolding and clearance. Among proteins affected by prenatal hypoxia are a key enzyme of the cholinergic system-acetylcholinesterase, and the amyloid precursor protein (APP), both of which have important roles in brain function. Disruption of their expression and metabolism caused by prenatal hypoxia can also result, apart from early cognitive dysfunctions, in development of neurodegeneration in later life. Another group of enzymes affected by prenatal hypoxia are peptidases involved in catabolism of neuropeptides, including amyloid-β peptide (Aβ). The decrease in the activity of neprilysin and other amyloid-degrading enzymes observed after prenatal hypoxia could result over the years in an Aβ clearance deficit and accumulation of its toxic species which cause neuronal cell death and development of neurodegeneration. Applying various approaches to restore expression of neuronal genes disrupted by prenatal hypoxia during postnatal development opens an avenue for therapeutic compensation of cognitive dysfunctions and prevention of Aβ accumulation in the aging brain and the model of prenatal hypoxia in rodents can be used as a reliable tool for assessment of their efficacy.

## Introduction

In recent years, a growing body of clinical, epidemiological and experimental studies testify to a crucial role of gestational factors in brain development and functioning in postnatal life, which increases its vulnerability to later development of neurodegenerative disorders including Parkinson's and Alzheimer's diseases (Faa et al., 2014). It has clearly been demonstrated that factors such as diet, infectious disease, drug administration, smoking and alcohol consumption, as well as persisting maternal stress, significantly affect fetal brain development and its function after birth (for review see Charil et al., 2010; Li et al., 2012; Monk et al., 2013; Donald et al., 2015; Gawałek and Sliwowska, 2015; Kohlmeier, 2015; Labouesse et al., 2015). The periods of prenatal development and early postnatal life are extremely important for formation of brain structures which will be involved in cognitive functions, including learning and memory, and shape life experience and character of individuals (Babenko et al., 2015; Desplats, 2015). Any disruptions in these periods could result in compromised neuronal networking and manifest themselves at different stages of postnatal life predisposing individuals with aging to development of neurodegenerative diseases. This raises an important question about the importance of early intervention for preventing neurodegeneration caused by pre- and perinatal pathologies. However, to achieve this goal, clinicians need a deeper understanding of the pathological changes caused by prenatal stress and how to attenuate them using various pharmacological and behavioral approaches developed from basic science. For this, studies of the effects of various pathological conditions during pre- and early postnatal development using animal models which reproduce various stages of human embryogenesis, especially in fetal brain development, are of particular importance (for review see Maccari et al., 2016).

Despite intensive research and accumulation of significant amounts of experimental data, the mechanisms underlying developmental deficits caused by prenatal pathologies are still not well understood. In recent years the concept of epigenetic programming of neurological disorders attempts to explain how prenatal stress via epigenetic alterations of the developmental programme in the fetal brain affects mental health in later life (Babenko et al., 2015). This concept suggests latent early-life associated regulation mechanisms (LEARn) induced by environmental agents in the prenatal or early postnatal period which might underlie development of neurodegenerative disorders, including late onset Alzheimer's disease (Maloney et al., 2012).

**Figure 1:**
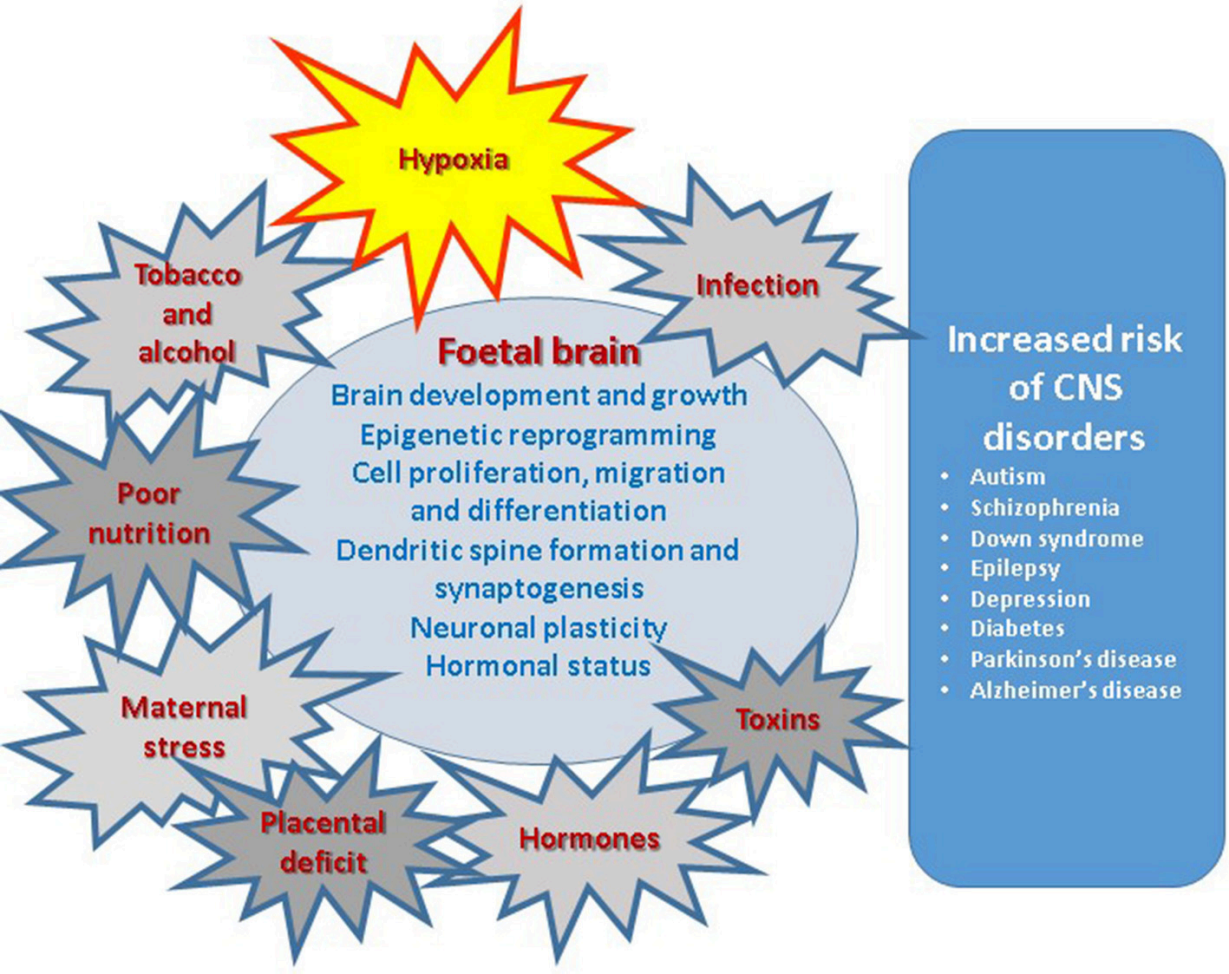
Prenatal factors affecting various aspects of brain development result in increased risk of central nervous system disorders in postnatal life.

In this review paper we will discuss various aspects of research accumulated to date, including our own multidisciplinary studies using the model of maternal hypoxia, with the aim to make a comprehensive analysis of the role of intrauterine hypoxia, in the development of neurodegeneration in later life as outlined in Figure 1. Since complications during pregnancy very often lead to insufficient oxygen supply to the fetus it is important to understand which changes they induce in the developing brain and how they can be prevented before, and compensated after, the birth. We have also summarized the main animal models of prenatal hypoxia developed to date. In some cases we also cite data obtained in the models of prenatal intrauterine ischaemia or neonatal hypoxia/ischaemia referring to the specific pathological conditions created in these models and their outcome for brain development. It is important to add that there are several animal models of neonatal hypoxia/ischaemia which have been extensively utilized and reviewed over the years (Vannucci et al., 1999; Vannucci and Hagberg, 2004; Patel et al., 2014; Rumajogee et al., 2016; Charriaut-Marlangue and Baud, 2018) and as such we have not specifically focused on this very important aspect of the research area.

## Response of the developing organism and brain to decreased oxygen content

Brain hypoxia is one of the most common complications resulting from impaired circulation and brain metabolism, which can affect animals and humans at various stages of life. Hypoxia elicits a wide range of physiological responses of the organism at the systemic, tissue and cellular levels. Although brain ischaemia and hypoxia are often considered to be of similar nature, ischaemia is usually characterized by a reduction or arrest of blood flow to certain brain areas causing irreversible neuronal destruction while hypoxia, on the contrary, leads to an increase in cerebral blood flow which might result both in permanent or reversible changes of neuronal functions depending on its severity (Miyamoto and Auer, 2000).

It is generally accepted that neuronal cells are more vulnerable to the effects of altered oxygen supply and, in particular, hypoxia than other types of cells (Haddad and Jiang, 1993; Erecinska and Silver, 2001). Indeed, already 5 s after arresting oxygen supply to the brain a significant functional impairment of the CNS can be observed followed by total loss of CNS functions and unconsciousness after 8–12 s of anoxia with resuscitation time for the brain not exceeding 10 min (Oechmichen and Meissner, 2006). This explains why hypoxic insults to the brain have very significant consequences and lead to severe pathologies.

It is well-known that prenatal hypoxia caused by abnormal pregnancy and labor leads to dramatic changes in the developmental profile and behavioral characteristics of animals and is one of the most common reasons for mental retardation and cognitive deficit in children (Nyakas et al., 1996). Lack of oxygen supply to the fetal brain can be caused by various maternal pathologies including infection (Gabrielli et al., 2012), vascular diseases and pre-eclampsia (for review see Meister et al., 2016). Animal studies also provide evidence that preeclampsia-like conditions leading to increased blood pressure, proteinuria, growth restriction and, in the most severe cases, intrauterine mortality also resulted in increased apoptotic cell death in the fetal brain with the most sensitive areas being the subventricular and pallidum zones (Pellicer et al., 2011). Despite the existence of various protective mechanisms during embryogenesis and at birth, exposure of the embryonic brain to maternal hypoxia results in a number of changes in its structure and functional properties (Gross et al., 1981; Vasilev et al., 2016a,b; Zhuravin et al., 2018).

### Oxygen sensing mechanisms

A decrease in O_2_ content in the surrounding atmosphere is detected by oxygen sensors represented by central and arterial chemoreceptors (for review see Sharp and Bernaudin, 2004). Central chemoreceptors are located in the medulla of the brainstem, near the respiratory centers. The term “arterial chemoreceptors” is generic and describes aortic body chemoreceptors and carotid body chemoreceptors. Activation of carotid bodies and aortic arch chemoreceptors stimulates neurotransmitter release pathways (for review see Kemp et al., 2002).

One of the principle enzymes involved in the oxygen sensing response in the carotid body is a neutral endopeptidase, neprilysin (NEP), which modifies the cellular response to hypoxia by hydrolysing substance P (Kumar et al., 2000). Prenatal hypoxia was shown to impair the response of the developing organism to hypoxia in adulthood by alterations of the catecholaminergic components of the chemoafferent pathway contributing to impaired postnatal respiratory behavior (Peyronnet et al., 2007). Various studies in animals and cell models demonstrate that hypoxia down-regulates expression of NEP which might contribute to these alterations (Nalivaeva et al., 2004, 2012; Fisk et al., 2007; Kerridge et al., 2015). The adult rats exposed to intermittent hypoxia in the neonatal period demonstrate augmented carotid body and adrenal chromaffin cell response to hypoxia and irregular breathing, which are associated with increased oxidative stress (Nanduri and Prabhakar, 2015).

In addition to chemoafferent pathways, oxygen sensing is also controlled by pulmonary neuroendocrine cells, which are mainly located in the neuroepithelial bodies, and exert chemosensitivity, which is especially important in early life (Caravagna and Seaborn, 2016). Apart from oxygen sensing, chemosensory response to olfactory and gustatory stimuli in early life play a very important role in the development of new-born organisms. However, the effects of prenatal hypoxia on development of these chemosensory systems have barely been studied. However, there are data reporting deficits in motor responses to olfactory stimuli in rabbits submitted to prenatal hypoxia (Tan et al., 2005), which might be linked to a decrease in NO-dependent signal transduction (Drobyshevsky et al., 2012). Interestingly, in healthy adult humans, hypoxia caused by mimicking high altitude oxygen content at 4,000 m above sea level resulted in reduced olfactory sensitivity and intensity (Huppertz et al., 2018).

### Tolerance to hypoxia

Although the fetus during development in the womb grows under the conditions of reduced oxygenation, to withstand the reduced oxygen supply its cells and organs have developed several compensatory responses to hypoxia in the process of evolution (for review see Giussani, 2016). However, these defense and tolerance systems are sometimes not sufficient for protecting the developing brain against acute or chronic reduction in oxygen supply caused by various pathological conditions (Gunn and Bennet, 2009).

The unifying theory of hypoxia tolerance at the molecular and metabolic levels was put forward by Hochachka and colleagues in the 1990s based on a comparative analysis of the reactions of cortical neurones and hepatocytes from high anoxia-tolerant and more hypoxia-sensitive systems (Hochachka et al., 1996). According to this hypothesis, the general mechanisms which are involved in the hypoxic response of cells include modulation of ATP-demands and ATP-supply pathways, suppression of protein synthesis, changes in metabolism and membrane function, which collectively affect ion transport and activity of cellular receptors and channels. In hypoxia-sensitive cells this translational arrest seems to be irreversible whereas, in the hypoxia-tolerant system, activation of the rescue mechanisms during an extended period of oxygen deficiency results in preferential expression of the same key proteins. Additionally, hypoxia-tolerant cells and tissues under hypoxic conditions use anaerobic metabolism, not for replenishing the energy deficits as in the sensitive cells, but for sustaining reduced energy turnover (Arthur et al., 1997).

The main difference between the reaction of liver and brain cortical cells of highly hypoxia-tolerant animals (e.g., turtle) to hypoxia is that in the hepatocytes the ATP demands for ion pumping are achieved by generalized “channel” arrest while the energy saving in the brain is mainly achieved by down-regulation of firing rates of synaptic transmission (so-called “spike arrest”) (Pamenter et al., 2011). More recently, this hypothesis has been further specified and the term of “synaptic arrest” introduced (Buck and Pamenter, 2018). Synaptic arrest happens mostly due to the down-regulation of excitatory amino acid (especially glutamate) release accompanied by increased release of inhibitory amino acids. This results in less pronounced (by 50%) metabolic suppression in brain cells than in the liver cells where metabolic rate is decreased down to 10% of the normoxic levels. In terms of energy expense it means that the ATP turnover rates in anoxic turtle neurons are higher than in the liver cells. These specific changes in the neuronal cells dictated by reduced oxygen supply are accompanied by altered functional expression of ion channels in various brain cell types (Peers, 2002). Alteration of channel expression as an adaptive reaction to prenatal hypoxia may contribute to development of various neuropathologies in later life, including AD, whose pathogenesis involves impairment of various types of ion channels (Hynd et al., 2004).

Unlike chronic hypoxia, exposure of organisms to repetitive episodes of transient mild hypoxia leads to development of brain hypoxic/ischaemic tolerance, the phenomenon termed hypoxic preconditioning (for review see Rybnikova and Samoilov, 2015). This preconditioning by mild hypoxia induces adaptive changes in the organism and the brain, effectively preparing them to sustain more severe hypoxic or ischaemic conditions. Although there are no extensive data on the effects of hypoxic preconditioning during the prenatal period, the available literature testifies to its preventive action against further hypoxic or ischaemic insults (Nalivaeva et al., 2004; Zhao and Zuo, 2005; Giusti and Fiszer de Plazas, 2012). In particular, prenatal hypoxic preconditioning was shown to reduce neuronal loss and apoptosis of brain cells after cerebral ischaemia in new-born rats which was iNOS-dependent (Zhao and Zuo, 2005). Our own data also suggest that mild hypoxic preconditioning protects some enzymes of amyloid metabolism from their decrease caused by severe prenatal hypoxia and ischaemia, and can be considered neuroprotective (Nalivaeva et al., 2004).

### Role of placenta in response to hypoxia

The damaging effect of hypoxia on the fetal brain can be compensated by various defense mechanisms in the placenta regulating blood supply to the fetus (Schneider, 2009) and allowing the brain to develop at the expense of other organs (Browne et al., 2015), although fetal circulatory redistribution does not necessarily spare the brain from harmful effects of hypoxia (Roza et al., 2008). Adaptive mechanisms found in fetal as well as neonatal tissues show striking similarities to survival strategies seen in mammals with a high tolerance to severe hypoxia like hibernators or deep-sea divers (Singer, 1999). The special ability of the mammalian fetus/neonate to tolerate a considerable degree of hypoxia in the perinatal period has been regarded as protection against hypoxic threats inherent in the birth process (Mortola, 1999). Increased tolerance to hypoxia, as such, is a result of various metabolic responses including “hypoxic hypometabolism” aimed at economizing oxygen consumption (Rohlicek et al., 1998).

In response to hypoxia and ischaemia, the placental-fetal unit initiates several response reactions aimed to allocate oxygen and nutrients preferentially to the fetus and increase its chance for survival (for review see Murray, 2012; Smith et al., 2016). Exposure to ischaemia-hypoxia leads to changes in metabolic and energy demand (Wheaton and Chandel, 2011) and angiogenesis (Ishimura et al., 2009) both in the placenta and fetus. In the developing brain it leads to decreased expression of proteins involved in cortical angiogenesis and reduced capillary density in the fetal brain (Cohen et al., 2014).

Another important protein in the placenta, namely the thyroxine and retinol transporter transthyretin (TTR), which plays an important role in fetal brain development (Makover et al., 1989; Chan et al., 2009), was also shown to be significantly upregulated by hypoxia (Patel et al., 2012). Although increased levels of this protein might be considered as a compensatory mechanism for stabilizing transport of hormones to the fetuses under hypoxic conditions, the data on increased levels of misfolded TTR aggregates in human trophoblast cells under hypoxic conditions suggest that hypoxia might also lead to its misfolding and aggregation and contribute to placental pathogenesis (Cheng et al., 2016). Indeed, increased levels of misfolded and oxidized forms of TTR have been reported in the amniotic fluid of pregnant women with preeclampsia that can serve as a predictor of developing pathology (Vascotto et al., 2007). Increased levels of TTR protein expression have also been found in the choroid plexus of rat pups subjected to prenatal hypoxia (Vasilev et al., 2018) which suggest its role in providing pathologically developing fetal brain with an increased supply of thyroxine and retinol.

### FETAL response to hypoxia

One of the important features of developing fetuses is that, under decreased oxygen supply, blood flow is drastically redistributed to the brain and heart from other organs increasing up to 90 and 240%, respectively, and this reaction is similar both in the pre-term and near-term fetuses (Richardson et al., 1996). In response to hypoxia the fetal brain also depresses its oxygen consumption via increased levels of adenosine acting on neuronal A1 receptors and vasodilatation through activation of A2 receptors on cerebral arteries (for review see Pearce, 2006). Apart from adenosine, hypoxia-induced release of nitric oxide also accounts for cerebral vasodilatation observed in the fetus (Cai et al., 1998). However, the reaction of fetal brain to acute and chronic hypoxia has different underlying mechanisms (Pearce, 2006).

Generally, at the cellular level, the initial response to hypoxia causes changes in expression of hypoxia-inducible factor (HIF), which regulates a number of genes, including practically all genes of the glycolytic pathway (for review see Semenza, 1998). It helps the organism to enhance its survival under hypoxic conditions by promoting erythropoiesis, angiogenesis and vasodilation. For example, hypoxia increases expression and secretion of the hormone erythropoietin, which improves systemic oxygen supply by enhancing the rate of erythrocyte formation, as well as transferrin, vascular endothelial growth factor (VEGF), leptin and other factors of angiogenesis and vascular tone (Chen et al., 2006). Other cell responses promote cellular survival by enhancing the expression of glycolytic enzymes, cell membrane glucose transporters including GLUT1, GLUT3, and other genes that tend to protect the cell from more severe oxygen deprivation (Vannucci et al., 1996). Other target genes for HIF-1 include enzymes of extracellular matrix metabolism (MMPs, plasminogen activator receptors and inhibitors), as well as factors of cell proliferation and apoptosis (Carmeliet et al., 1998). Deficiency of HIF-1 in the maternal organism leads to placental abnormalities which makes the fetus vulnerable to oxygen deprivation after mid-gestation (Kenchegowda et al., 2017). During embryonic and postnatal brain development, HIFs and specific HIF target genes are widely involved in early and highly active maturation processes via modulation of cell proliferation and differentiation (Trollmann and Gassmann, 2009). Although these genetic changes aim to protect neural tissue from ischaemia and hypoxia during development, their effects may persist and alter susceptibility for neurodegeneration later in life.

Prenatal hypoxia was shown to amend expression of GLUT4 and HIF-1α gene expression in fetal rat brain with different modality depending on the embryonic stage (Royer et al., 2000). At E14, exclusively, gestational hypoxia in rats was found to increase mRNA transcript levels of HIF-1α, GLUT3, GLUT, thyroid hormone receptors (TR) TRα2 and TRβ1 genes. However, hypoxia on E19 does not result in any response of HIF-1α and GLUT3 genes indicating differences in adaptation to hypoxia at these periods of development. This might explain the different impacts of prenatal hypoxia at different stages of rat embryogenesis on the development of cognitive functions in postnatal life as observed in our experimental model of prenatal hypoxia in the rat (Dubrovskaya and Zhuravin, 2010).

Microglia are now considered a very important safeguard of the healthy brain and their activation was shown to play an important role in the response of the neonatal brain to hypoxic-ischaemic injury (for review see Mallard et al., 2018). Prenatal hypoxia and ischaemia have also been shown to activate microglia in the developing brain. Thus, ischaemic insult in rats on E18 resulted in an increased number of microglial cells (Robinson et al., 2005). Moreover, it was shown that an insufficient oxygen supply leads to changes in the inflammatory/immune response in the fetal brain which can be detected in early postnatal life by an increased content of the pro-inflammatory cytokines IL-6, IL-10, and TNF-α in the CSF of new-born infants (Ellison et al., 2005). Furthermore, in a rat model of global fetal and perinatal asphyxia, changes in cytokine and ceramide metabolism genes in the prefrontal cortex, hippocampus and caudate-putamen were observed even at the age of 8 months after birth (Vlassaks et al., 2013). It was also shown that microglial activation in the brain of 1 day old rat pups subjected to hypoxia requires Notch signaling and activation of the NF-κB pathway (Yao et al., 2013).

## Epigenetic consequences of prenatal hypoxia

In the last decade a significant amount of studies have been focused on identification of the genetic and epigenetic factors that might link the effects of pre- and perinatal hypoxia and ischaemia with the risk of development of neurodegenerative disorders in later life. Since it has been shown that a number of specific genes in human cortex change their expression during fetal and early postnatal development, and that this pattern of gene expression is mirrored in aging and in neurodegeneration (Colantuoni et al., 2011), it is reasonable to expect that prenatal environment might, to various extents, affect these processes. One gene highly important in early brain development, which then changes in adult neurogenesis, is the RE1-silencing transcription factor, REST (Lunyak et al., 2002; Otto et al., 2007). Changes in expression of REST have been shown to correlate with mild cognitive disorders and Alzheimer's disease (Nho et al., 2015). Dysregulation of REST has also been implicated in the pathogenesis of Huntington disease and Down syndrome (Bahn et al., 2002; Buckley et al., 2010) and was shown to be involved in stress resistance in aging and Alzheimer's disease (Lu et al., 2014). This factor protects genomic integrity during embryonic development (Nechiporuk et al., 2016) and is induced in hypoxia leading to the changes in approximately 20% of hypoxia-repressed genes (Cavadas et al., 2016).

Although changes in the expression of hypoxia-responsive genes during prenatal development have an adaptive character, their epigenetic modifications may result in neurodevelopmental vulnerability and, in particular, underlie the attention deficit/hyperactivity disorders (ADHD) in children (Smith et al., 2016). Indeed, it was shown that SNP polymorphisms in angiogenic, neurotrophic and inflammatory genes are involved in response to an adverse prenatal environment and correlate with the severity of ADHD in children (Smith et al., 2014).

The epigenetic mechanisms which can be affected by prenatal hypoxia and ischaemia involve such processes as DNA methylation and histone modifications which regulate chromatin folding and gene activation or silencing (for review see Johnson and Barton, 2007) and brain development (Kato and Iwamoto, 2014; Tapias and Wang, 2017). Moreover, identification of a novel class of histone demethylases as true dioxygenases suggests that chromatin can act as an oxygen sensor coordinating cellular response to hypoxia (Melvin and Rocha, 2012).

### Chromatin modifications

Although not sufficiently studied existing data suggest direct involvement of chromatin modification at the levels of histone acetylation and DNA methylation in the fetal response to hypoxia. Some examples are listed below.

At the organ-specific level in a lamb model of high-altitude long-term prenatal hypoxia it was shown that fetal pulmonary arteries have reduced levels of global histone 4 acetylation and DNA methylation, accompanied by the loss of the cyclin-dependent kinase inhibitor p21 which were linked to development of pulmonary arterial remodeling and pulmonary hypertension of the new-born (Yang et al., 2012).

Analysis of expression of glucose 6-phosphatase (G6Pase), which is involved in gluconeogenesis, in a rat model of maternal hypoxia (11.5% atmospheric oxygen from E15 to E21) demonstrates that in male offspring decreased hepatic G6Pase mRNA and protein levels correlated with increased methylation of histone H3 surrounding the *G6Pase* promoter (Osumek et al., 2014).

Oxygen levels have also been shown to regulate epigenetically the fate of brain mid-gestational neural precursor cells via HIF1α-Notch signaling interaction and DNA demethylation of astrocytic genes (Mutoh et al., 2012). In normally developing brain, lower oxygen tension characteristic of the embryonic brain (below 5%) promotes differentiation of mid-gestational neuronal precursor cells into astrocytes via activation of the Notch-signaling pathway and up-regulation of transcription factor NFIA. This leads to DNA demethylation of such astrocyte specific genes as *gfap* and *S100*β. However, under normoxic conditions (21% O_2_) these processes are inhibited. Considering these data, it is reasonable to suggest that decreased oxygen levels will have an even stronger promoting effect on astrocyte differentiation in developing brain resulting in a decreased neurone-astrocyte ratio. Indeed, in fetal guinea pigs subjected to maternal hypoxia (10.5% O_2_ in the air, E52-62) the density of NeuN-immunoreactive neurons in the fetal CA1 hippocampal area was subsequently decreased (Blutstein et al., 2013). Additional treatment with nicotine in the same model significantly increased the number of astrocytes in the fetal hippocampus and resulted in reductions of both GFAP- and NeuN-positive cells in the CA1 in adulthood. It is important to note that comparing epigenetic landscapes of neuronal and glial cell genomes in normally and pathologically developing brains might in the future provide a powerful tool for creating genetic maps of normally developing and aging brain and provide clues for their changes in neurodegeneration (Mitchell et al., 2014).

Decreased expression of glucocorticoid receptors in developing rat brain caused by maternal hypoxia (10.5% O_2_, E15-21) was also shown to involve increased DNA methylation in the area of exons 17 and 111. This decreased binding of the transcriptional factors Egr-1 and Sp1 to the promoters led to reduced levels of exon 17 and 111 mRNA variants (Gonzalez-Rodriguez et al., 2014).

Changes in DNA demethylation of the corticotropin-releasing hormone *Crhr1* gene was also observed in the hypothalamus of male offspring of Sprague–Dawley rats subjected to intermittent hypoxia during whole pregnancy (10.8% O_2_, for 4 h per day, E1–E21). These data suggest the existence of hypoxia-triggered male-sex-dependent regulation of the *Crhr1* gene via demethylation at CpG sites in the promoter region leading to development of anxiety-like behavior in adulthood (Wang et al., 2013).

Demethylation of genomic DNA and a decreased level of DNA methyltransferase 3b expression *in vivo* was shown to result in elevated levels of APP, β- and γ-secretases in 3-month-old offspring of transgenic mice (APP^SWE^/PS1ΔE9) exposed to an intermittent hypoxic environment (6 h/day) for 30 days. On the contrary, overexpression of DNA methyltransferase 3b reduced the levels of these proteins in *in vitro* cell models (Liu et al., 2016). Such alterations in expression of the Alzheimer's disease-related genes of hypoxic mice were accompanied by learning and memory deficits in later life.

Increased DNA methylation of a specific site in the BDNF gene harboring functional SNP rs6265 for the Val(66)Met allele predisposing the ValVal individuals to impaired working memory and increased risk of a schizophrenic phenotype, was reported in the peripheral blood mononuclear cells of children with a history of obstetric complications during labor-delivery (Ursini et al., 2016). Taking into account that brain DNA methylation in children cannot be studied *in vivo*, blood cell analysis might provide a non-invasive and useful approach for detecting epigenetic abnormalities caused by pathologies during pregnancy and labor.

### Micro RNA

Accumulating data also support the involvement of a distinct class of small noncoding microRNAs (miRNAs) in post-transcriptional regulation of target genes in response to hypoxia (for review see Nguyen et al., 2013). Since the machinery of miRNA production and maturation depends on oxygen supply to the cells (Ho et al., 2012) and regulation of HIF expression, in turn, depends on miRNA species (Tanaka et al., 2013; Liu et al., 2015), in particular, on miR-17-92 (Taguchi et al., 2008), it is reasonable to suggest that miRNAs might also participate in the response of neuronal cells to hypoxia. Indeed, there are studies suggesting that hypoxia alters miRNA expression in rat cortical pericytes (Truettner et al., 2013) and hippocampus (Gao et al., 2017) which leads to cognitive dysfunction.

A reverse correlation between expression of miRNA species and REST in neuronal cells in response to hypoxia also suggests a regulatory role of reduced oxygen supply in maintaining neuronal miRNA profiles (Liang et al., 2014). Microarray analysis of neural progenitor cells has shown that 15 microRNAs were up-regulated at least 3-fold and 11 were down-regulated under hypoxic conditions with specifically increased expression of miR-210 regulated by HIF-1α (Liu W. et al., 2011). In murine embryonic brain cortices during hypoxia-induced neuronal apoptosis, the miR-23b-27b cluster was also found to be downregulated depending on the transcription factor c-Myc (Chen et al., 2015). Taking into account that a number of recent studies have identified the role of miRNAs in neurodegenerative disorders, including AD, PD and Huntington's disease (Maciotta et al., 2013; da Silva et al., 2016; Salta and De Strooper, 2017), changes in the miRNA pattern in developing brain after prenatal hypoxia will certainly result in a neurodegeneration-prone phenotype in later life. Quantitative analysis of hypoxia-regulated miRNAs in the maternal blood may provide a tool for identifying risk of fetal hypoxia (Whitehead et al., 2013). Based on the recent data on aberrant hypoxia signaling due to pre-eclampsia, which involves deregulated expression of miR455, development of a non-invasive test based on miRNA analysis in the blood of pregnant women might be beneficial for early diagnostics of pre-eclampsia-related pathological changes in the placenta and fetal development (Lalevée et al., 2014).

### Post-translational modifications of proteins

Post-translational modifications of proteins substantially affect their normal properties and hence metabolism (Nalivaeva and Turner, 2001). The accumulation of abnormally modified and folded proteins can lead to development of various neurodegenerative disorders including Alzheimer's, Parkinson's, and Huntington's diseases (Ren et al., 2014). Since proper protein folding and removal of misfolded proteins is an important part of proper brain development, analysis of the effects of prenatal hypoxia on the status of protein posttranslational modifications and aggregation in the brain is an important indicator of its healthy development.

Although this area of research is still not sufficiently developed, there are indications that hypoxia increases protein ubiquitination detected even 6 months after perinatal hypoxia (Capani et al., 2009; Grimaldi et al., 2012). Hypoxia also leads to activation of chaperone-mediated autophagy (Dohi et al., 2012) and it is reasonable to expect that autophagy mechanisms might be disrupted after prenatal hypoxic stress leading to accumulation of misfolded proteins, ER stress and metabolic syndrome in later life. As such, one of the neuroprotective strategies for treatment of prenatal hypoxia related brain disorders in postnatal life might be activation of autophagy-related cellular mechanisms (for review see Herrera et al., 2018).

## Risk of development of neurodegenerative disorders

In this connection it is important to mention the Developmental Origins of Health and Disease hypothesis (DOHaD) based on the theory of fetal programming originally outlined for coronary heart diseases (Barker, 2007) but then extended to mental health disorders (Swanson and Wadhwa, 2008; O'Donnell and Meaney, 2017). The supporters of this hypothesis raise the question of the importance of epigenetic reprogramming during embryonic development in response to various adverse environmental factors, which makes the organism vulnerable for development of disease in later life. They also stress the importance of studying the fetal origin of mental health.

As already mentioned above and will be discussed further, accumulated research to date suggest a link between prenatal hypoxia and increased risk of development of Alzheimer's disease pathology in later life (Nalivaeva et al., 2012; Zhang et al., 2013; Wang et al., 2014; Liu et al., 2016). Although there have been experimental attempts to link prenatal hypoxia and other pathological factors in early life with the pathogenesis of Parkinson's disease, there is no direct evidence of such a correlation (Gardener et al., 2010). Nevertheless, there are reports that prenatal hypoxia results in selective and long-lasting impairments in the dopaminergic systems that can be detected even in adulthood (Burke et al., 1992; Chen et al., 1997). A search for other environmental factors which might affect development of the nigrostriatal dopaminergic system of the brain during embryogenesis revealed that exposure to some toxicant pesticides can predispose to Parkinson's disease in the male offspring (Barlow et al., 2004). Exposure to neurotoxins, e.g., bacterial lipopolysaccharides, during critical developmental periods in pregnancy might also be a risk factor since it leads to a decreased number of dopamine neurones in the brain of the offspring (Carvey et al., 2003).

Stronger evidence suggests a link between prenatal hypoxia and the development of schizophrenia (Van Erp et al., 2002; Howell and Pillai, 2014). Studies in rodent models indicate that chronic hypoxia leads to anatomical abnormalities often observed in schizophrenic patients (Asami et al., 2012; Andreasen et al., 2013). Moreover, rodent models have been found useful for investigating sex-related susceptibility to brain damage by neonatal hypoxia and development of schizophrenia (Mayoral et al., 2009).

## Models of prenatal hypoxia

For better understanding the role of prenatal hypoxia in fetal pre- and post-natal development, various animal models have been employed over the span of several decades (for review see Rees et al., 2008). Some have been developed based on restriction of uterine blood flow to the fetus using, for example, vascular occlusion in pregnant sheep which clearly demonstrated that prolonged hypoxia, induced by placental insufficiency of differing severity and duration, causes changes in fetal brain structure (Clark et al., 1982; Rees et al., 1998). Using this model it was demonstrated that transient hypoxia also affected morphology and functions of various types of neuronal cells and impaired development of neural processes and connections, involving the subplate neurons, which regulate brain development and play a critical role in establishing cortical connections to other brain regions (McClendon et al., 2017). Moreover, it was found that subplate neurons of the sheep fetus are surprisingly resistant to hypoxia and acquire some chronic structural and functional changes that might be beneficial to neuronal survival and brain connectivity in postnatal life. The authors have clearly demonstrated that there are species-associated differences in the response to hypoxia between rodents and sheep that should be taken into account when interpreting and comparing the experimental data obtained in different animal models. The sheep models to date provide a platform for studying various physiological changes in the developing brain under restricted oxygen supply including electroencephalography (Abbasi et al., 2017).

Another model of prenatal hypoxia in sheep applied reduced oxygen content in a maternal ventilated gas mixture by partially replacing it with nitrogen (Tchirikov et al., 2011). Using this approach it was demonstrated that acute maternal hypoxia results in reduced placental blood perfusion in the hypoxemic fetuses and lower fetal pH and pO_2_ compared to normoxic fetuses.

Among medium-size animals convenient species for modeling prenatal hypoxia are rabbits (Gingras and Long, 1988; Buser et al., 2010) and guinea-pigs (Mishra et al., 1988). For animals of this size an environmental chamber with reduced oxygen content can be used in which pregnant females are kept under lower oxygen content of different severity at various stages of pregnancy for the required duration (Oh et al., 2008). The experiments in rabbits have shown that prenatal hypoxia results in a wide spectrum of biochemical changes in brain tissue of the offspring (Gingras et al., 1995), including reorganization of the white matter, brain morphology and functions. These studies have significant implications for understanding the role of prenatal hypoxia for prematurity and cerebral palsy in children (Coq et al., 2016). The works of Mishra and colleagues utilizing the guinea pig model of prenatal hypoxia over a decade are summarized in a review article (Mishra and Delivoria-Papadopoulos, 1999) which underlines the enhanced susceptibility of the brain to hypoxia in the process of fetal development and increase in the demands of neuronal cells to oxygen supply. More recent studies in the guinea pig model provide detailed characterization of the changes in fetal brain energetics caused by chronic prenatal hypoxia (Wang et al., 2016). They also confirm that prenatal hypoxia affects various systems of the developing brain resulting in a decreased number of neurons in the cerebral cortex and dentate gyrus of the fetus correlated with reduced levels of BDNF (Chung et al., 2014, 2015).

Recently, development of transgenic mice modeling various neurodegenerative conditions has provided researchers with useful tools for studying the effects of prenatal hypoxia on acceleration of development of brain pathologies, including Alzheimer's disease (Zhang et al., 2013; Rueda-Clausen et al., 2014). These models allow investigation of detailed molecular mechanisms underlying development of brain pathology in the prenatal period and a search for possible therapies (Wang et al., 2014).

However, rats are still the most common experimental animals for modeling prenatal hypoxia and studying its short- and long-term effects on various aspects of brain development and characteristics (Golan and Huleihel, 2006). In rats, prenatal hypoxia can be achieved via reducing oxygen content in a chamber, where pregnant rats are housed and replacing it with an inert gas (commonly, nitrogen) (Gross et al., 1981; Zhuravin et al., 2004). Hypoxia can also be produced in a chamber with hypobaric conditions, which model high altitude hypoxia (Tyulkova et al., 2011) or by exposure of pregnant rats to chronic mild carbon monoxide concentrations (Beltran-Parrazal et al., 2010). Some chemical approaches e.g., administration of sodium nitrite (Nyakas et al., 1994) or a nitric oxide synthase inhibitor N(ω)-nitro-L-arginine methyl ester (L-NAME) can also be used (Pellicer et al., 2011) as well as surgical procedures restricting blood flow to the fetuses e.g., umbilical cord occlusions (Smotherman and Robinson, 1988) or unilateral uterine artery ligation (Magal et al., 1990; Tashima et al., 2001).

The list of animal models for studying the effects of hypoxia on the developing brain cannot be completed without mentioning an important area of research in peri- and neonatal hypoxia and ischaemia. These conditions mimic various pathologies to which the fetus might be subjected around the time of birth leading to neonatal hypoxic-ischaemic encephalopathy. Most of these models are based on the classic Rice-Vannucci model in which rat pups on P7 undergo unilateral ligation of the common carotid artery with subsequent exposure to 8% oxygen in the breathing air for several hours (Rice et al., 1981). Taking into account that the rat brain continues to mature during the first month after birth and at P7 is morphologically similar to the human fetal brain at 32–34 weeks of gestation, this model provides significant insights into how hypoxia and/or ischaemia affect immature brain cells. However, it is important to note that, after birth, brain metabolism in rat pups undergoes adaptation to extra-uterine oxygenation which might change the reaction of neurones to hypoxia-ischaemia. Nevertheless, utilization and modifications of this model over the decades have produced a significant amount of important information on the cellular mechanisms involved in reaction of the brain to hypoxia and have allowed development of therapeutic avenues for treatment of encephalopathy in children (Gancia and Pomero, 2012; Patel et al., 2014; Rumajogee et al., 2016; Edwards et al., 2017).

## Prenatal hypoxia in rats

**Figure 2:**
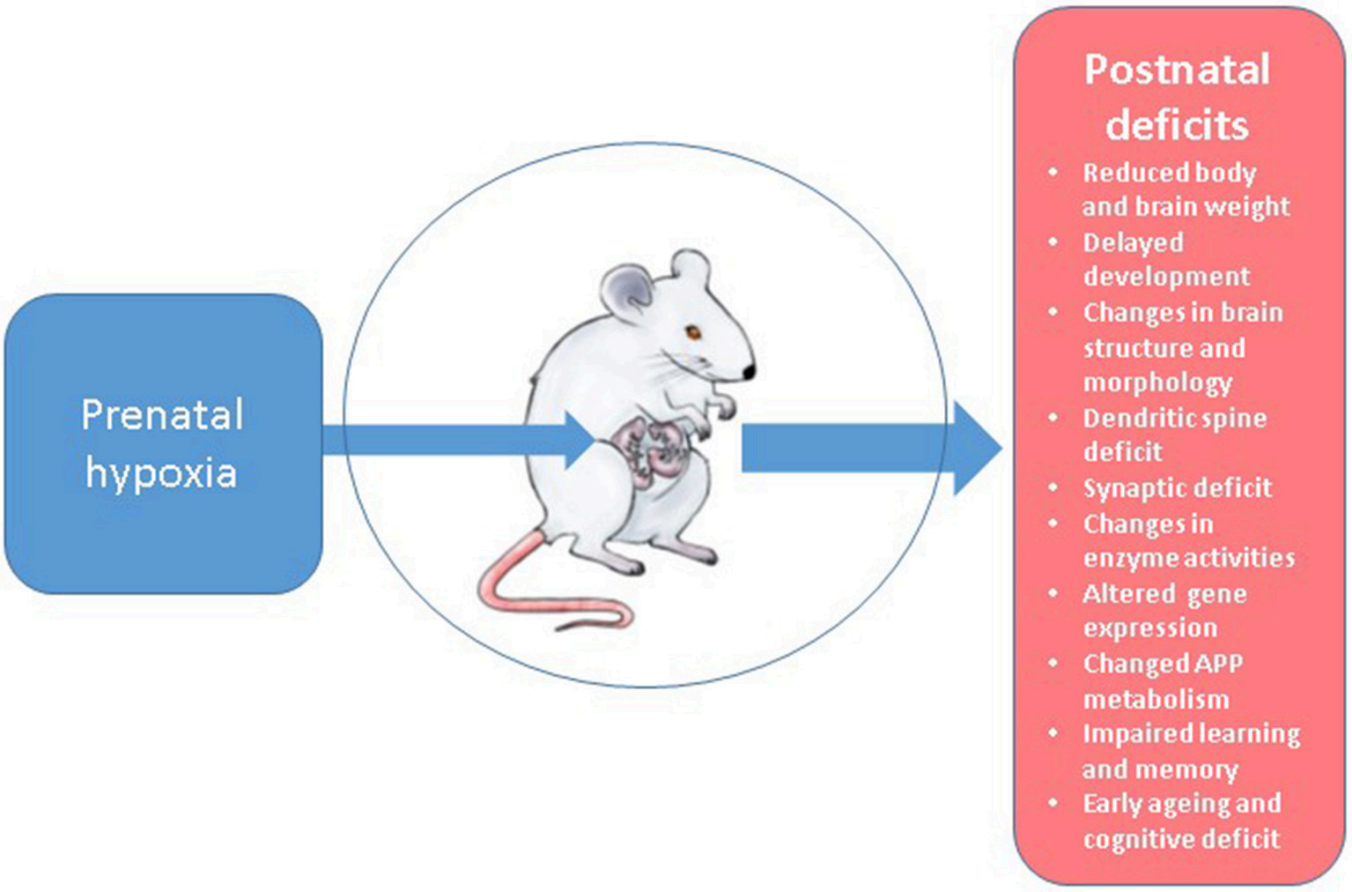
Postnatal deficits observed in the rat model of prenatal hypoxia.

In our studies for more than two decades we have developed and intensively utilized a model of normobaric hypoxia using laboratory Wistar rats at various days of pregnancy which is described in detail in our early work (Zhuravin, 2002; Lavreneva et al., 2003). For this we use a 100 L chamber supplied with gas analysis equipment, thermoregulation, and facility for removal of excess CO_2_. The hypoxic conditions are achieved by replacing oxygen with nitrogen down to 7% O_2_ concentration (or other desired level) during 10 min and then remaining at this level for 3 h. This paradigm provides a reliable and reproducible setting for maintaining hypoxic conditions and obtaining the material for further experiments either from the fetuses or rat pups during different stages of their postnatal development. The detailed analysis of the data obtained in these studies has recently been reviewed in Zhuravin et al. (2018). Below we shall discuss the main effects of prenatal hypoxia on rat brain anatomical, biochemical and functional properties (Figure 2) comparing the results of our studies with the data of other research groups employing different hypoxia paradigms.

### Structural changes in rat brain after prenatal hypoxia

There is a significant amount of data demonstrating that prenatal hypoxia results in a set of physiological changes in rat embryos leading to functional and behavioral changes in the postnatal period including reduced body weight of newborn pups (Gross et al., 1981; Olivier et al., 2005; Dubrovskaya and Zhuravin, 2010). Although some authors have not observed significant changes in brain weight of rat pups after prenatal hypoxia (Gross et al., 1981; Liu Z. H. et al., 2011) they have reported changes in the brain to body weight ratio (Liu Z. H. et al., 2011) as well as DNA/protein ratio (Gross et al., 1981). In the experiments with chronic prenatal hypoxia (10.5% O_2_, E4-E21) the decreased brain weight has been reported both in the fetuses and 6-week old offspring (Wei et al., 2016). However, other authors reported an increased brain weight in male offspring of Sprague-Dawley rats, subjected to maternal hypoxia (10.5% oxygen) on gestational day 21 (Zhang et al., 2016).

Obstructive sleep apnoea during pregnancy, and especially in late gestation, is a rather common complication in women. Intermittent hypoxia, to which the fetus is subjected during apnoea episodes, induces metabolic dysfunction which can be detected as increased body weight and higher adiposity index in adult male offspring. This suggests differential sex-dependent effects of the condition on expression of fetal genes (Khalyfa et al., 2017).

The major anatomical and structural alterations in rat brain after prenatal hypoxia are manifested at the level of the cellular composition of various brain structures (the cortex, hippocampus, striatum, cerebellum, etc.), including degeneration of neuronal cells, gliosis and apoptosis (Rees and Inder, 2005; Golan and Huleihel, 2006; Zhuravin et al., 2006; Liu Z. H. et al., 2011; Wang et al., 2017). Increased levels of apoptosis in rat brain after hypoxia correlated with upregulation of caspases, in particular of active caspase-3, which contributed to alteration in neuronal composition of different cortical layers (Vasilev et al., 2016a). Activation of apoptotic events caused by perinatal hypoxia modeling birth asphyxia was also shown in the cortex and CA1 area of the hippocampus in rat pups during the first 2 weeks after the insult resulting in reduced cell density and the accumulation of cells with nuclear fragmentation specific for apoptosis (Daval and Vert, 2004).

Importantly, it was also shown that prenatal hypoxia affects the cells in brain neurogenic zones and, in particular, the levels of expression of the protein paired box 6 (Pax6) which plays an important role in neurogenesis, cell proliferation, differentiation and survival during the development of the central nervous system (Simpson and Price, 2002). Although in the fetuses subjected to prenatal hypoxia the levels of Pax6 were increased in the subventricular zone and subgranular zone of the hippocampal dentate gyrus, they were significantly decreased in the cerebral cortex (So et al., 2017). This finding correlates with the reduced number of neuronal cells in rat cortex during the first month of postnatal life observed in our studies (Vasiliev et al., 2008).

Using light and electron microscopic techniques it was demonstrated that prenatal hypoxia caused a delay in differentiation of neurones and formation of synaptic contacts in rat neuropil as well as affecting myelination of nerve fibers at the ultra-structural levels both in the neocortex and basal ganglia (Zhuravin et al., 2006; Vasiliev et al., 2008; Vasil'ev et al., 2010). In particular, on postnatal days P10-30 in the brain cortex there was a significant decrease in the total number of pyramidal neurones (Vasiliev et al., 2008). However, this decrease was observed only during the first month of rat postnatal development and only in the group subjected to prenatal hypoxia on E14, but not on E18 (Dubrovskaya and Zhuravin, 2010; Vasilev et al., 2016b). Changes in cell composition have also been observed in the dorsal hippocampus of hypoxic rats, especially in the CA1 with increased number of neurones possessing retracted apical dendrites (Zhuravin et al., 2009a).

The effects of prenatal hypoxia were more profound when it was applied at mid rather than late gestation and became less apparent with development of rats in the postnatal period (Nyakas et al., 1996; Dubrovskaya and Zhuravin, 2010). Because formation of main brain anatomical architecture starts on embryonic day 12 (E12) and the precursors of cortical and striatal neurons actively proliferate on E14 and of the hippocampal neurons on E15 (Rice and Barone, 2000), the timing of hypoxia or other insults determines their impact on cellular composition and structure of specific brain regions and therefore affects formation of the physiological functions related to them. For example, prenatal hypoxia on E14 and E18 resulted in different outcomes of neuronal migration into the cortical layers of rat cortex and performance of behavioral tasks in postnatal life (Vasilev et al., 2016b).

### Changes in development of brain functions

There are many tests available to assess the development of brain integrity in rats at very early stages after birth including habituation, exploratory behavior, reactivity and motor coordination. The most commonly employed tests include the body-righting reflex, negative geotaxis, placing, homing, head elevation, ascending on a wire mesh, which are extensively reviewed in Rice and Barone (2000). In our model of prenatal hypoxia in rats we also observed a delay in pup maturation (reduced body weight during the first month of life, delayed eye opening time and the onset of separation of the external ear from the skin of the head) and development of various sensory-motor reactions including the body-righting reflex, negative geotaxis, forelimb placing reaction, maintenance of balance on a rotating grid etc (Dubrovskaya and Zhuravin, 2010). Some developmental features such as later separation of the external ear from the scull, the forepaw-placing reaction and the whisker placing reaction were found to be delayed only after prenatal hypoxia on E14 and not on E18 (Dubrovskaya and Zhuravin, 2010; Vasilev et al., 2016b).

Although the deficit in innate motor reactions of new-born rats after prenatal hypoxia, observed in our studies, becomes less pronounced with pup development during the first month of postnatal life, execution of more skilful movements, e.g., reaching and pushing, and learning new motor reflexes were still compromised in adulthood (Zhuravin et al., 2002). This correlates with the observations that motor and coordination abilities remained partially impaired in the old rats subjected to prenatal hypoxia, especially under high oxygen demand (Jänicke and Coper, 1994). Some authors link the cause of motor deficit observed after prenatal hypoxia with a failure in the migration and maturation of oligodendroglial progenitor cells causing delay of myelination in the cerebellum (Barradas et al., 2016).

Unlike motor functions which are practically compensated and restored during the first month of pups' development, the cognitive deficits caused by prenatal hypoxia on E14 or E18 remain detectable at all tested stages of postnatal life. For example, various types of rat memory (working, short and long-term memory) assessed by 8-arm maze and novel object recognition were compromised and correlated with the structural changes observed in the hippocampus (Zhuravin et al., 2011; Nalivaeva et al., 2012; Cunha-Rodrigues et al., 2018). Some studies link working memory impairment observed both in juvenile and adult rats subjected to prenatal hypobaric hypoxia with increased levels of phosphatidylinositol 4,5-diphosphates and phosphatidylinositol 4-phosphates in the hippocampus and upregulated expression of the type 1 inositol 1,4,5-trisphosphate receptor (IP3R1) (Tyul'kova et al., 2015). Maternal hypoxia on days 15–21 was shown to result in increased activity of metalloproteinases and significant cell death in the hippocampus of rat pups on days 0–7 after birth, which correlated with worsened development of their neurobehavioral functions (Tong et al., 2010). On the other hand learning deficits in adult rats subjected to prenatal hypoxia was shown to correlate with a significant reduction in the number of neurones positive to the polysialylated markers in the dentate granular zone of the hippocampus (Foley et al., 2005).

Prenatal transient systemic hypoxia-ischaemia created in Sprague-Dawley rats by occlusion of uterine arteries for 60 min on E18 has recently been reported to cause a sustained motor deficit and poor social interaction in young adult rats, which were accompanied by impaired white matter microstructure and diffusion abnormalities in the hippocampus, striatum and thalamus (Robinson et al., 2018). In a similar model of prenatal hypoxia on E18 adult rat offspring also demonstrated increased anxiety behavior and reduced spatial exploration and deficit in habituation memory (Sab et al., 2013). Prenatal ischaemia induced by unilateral ligation of the uterine artery on E17 was also shown to induce motor hyperactivity and deficits in information encoding, and short- and long-term memory in adult offspring (P40 to P80) although no impairments in spatial learning or working memory were observed when animals were tested in the Morris water maze (Delcour et al., 2012).

According to our data, rats subjected to prenatal hypoxia demonstrate reduced ability to learn new instrumental reflexes. Thus, on postnatal days 20-30 the number of rats in the experimental groups capable of learning to push a piston inside a narrow tube was 30% lower compared to the control group and at the age of 3 months, the number of hypoxic rats capable of learning this reflex for a certain duration was 40% lower than in the control group (Zhuravin et al., 2002). Analysing the ability of rats to remember the learnt task after a 5 week interval it was found that control rats were able to remember the learnt duration of the reinforced movements while hypoxic rats returned to the level before training, which implies a significant memory deficit caused by prenatal hypoxia.

Impaired learning abilities of rats were also reported in other paradigms of prenatal hypoxia. Thus, a 30-min hypoxic insult by complete clamping of the uterine vasculature on E17 was found to impair spatial memory in the Morris water maze and caused learning deficits in the passive avoidance test during the first month of development (Cai et al., 1999). These abnormalities the authors linked to the reduction in NOS expression and activity in the affected brain areas. On the contrary, gestational intermittent hypoxia induced by computer-controlled exposure of pregnant Sprague-Dawley rats either to room air or to 10% O_2_ alternately every 90 seconds starting on E5 until delivery did not result in any changes in acquisition and retention of a spatial memory both at 1 and 4 months of age (Gozal et al., 2003). This outcome, however, might be attributed to the development of tolerance to hypoxia in the fetal brain caused by repeated short episodes of maternal hypoxia.

### Synaptic plasticity

Existing literature suggests that impaired brain functions caused by prenatal hypoxia are related to impaired neurotransmitter circuits and synaptic plasticity (Herlenius and Lagercrantz, 2004; Barradas et al., 2016; McClendon et al., 2017). In rats submitted to prenatal hypoxia on E14 we have also observed a significant reduction in the number of synaptopodin-positive dendritic spines (Zhuravin et al., 2011; Vasilev et al., 2016b) which are fundamental for the formation of synaptic contacts and memory (Martin et al., 2000; Zito et al., 2009; Segal, 2010). The decrease in the number of synaptopodin-positive dendritic spines was particularly evident in the molecular layer of the neocortex and in the CA1 area of the hippocampus which correlated with impaired working memory (Zhuravin et al., 2009b). This decrease in the number of labile dendritic spines in the CA1 area of the hippocampus might be related to the changes in the entorhinal cortex which, in humans, is considered to be the earliest event in the development of Alzheimer's disease (Killiany et al., 2002). Damage in the medial and lateral entorhinal cortices correlating with impaired memory have indeed been reported in adult rats subjected to prenatal hypoxia on E17 (Delcour et al., 2012). The reduction of the number of synaptopodin-positive spines along with decreased ability for learning is also observed in normally aging rats, which could be one of the reasons for cognitive decline related to advanced age, and in the sporadic form of Alzheimer's disease (Zhuravin et al., 2011; Arnold et al., 2013).

The mechanisms of impairment of neuronal interactions caused by prenatal hypoxia in rat brain are more complex and do not involve only the changes in the number of dendritic spines and neuronal contacts but also result in disruption in the development of various mediator systems in the postnatal period (Nyakas et al., 1994; Gerstein et al., 2005; Tyulkova et al., 2011). As we have also observed, prenatal hypoxia on E14 resulted in a decrease in the number of VAChT-positive cholinergic terminals which form synapses on the bodies of the pyramidal neurones in layers V-VI of the parietal cortex. On the other hand, the EAAT levels were found to be much higher in hypoxic animals resulting in spontaneous epileptogenic activity and increased kindling in response to pharmacological agents and other external stimuli (Zhuravin et al., 2018) and even a weak electric shock could induced seizure episodes in 1.5 years old rats subjected to prenatal hypoxia on E14 with more pronounced average duration than in control animals (Kalinina et al., 2015).

### Changes at the molecular level

Structural and functional changes in rat brain after prenatal hypoxia are underlined by significant alterations in its biochemical characteristics including various classes of molecules (nucleic acids, proteins and lipids) and metabolic pathways (Gross et al., 1981; White and Lawson, 1997; Peyronnet et al., 2000; Beltran-Parrazal et al., 2010; Camm et al., 2011). For example, acute prenatal hypoxia on E14 affects activities of the different forms (cytosolic, membrane-bound, and soluble) of acetyl- and butyryl-cholinesterases (AChE and BChE) in the sensorimotor cortex detected at various stages of postnatal ontogenesis (Lavreneva et al., 2003; Kochkina et al., 2015). The increase in brain BChE activity might have a compensatory effect on the stress response of the brain due to the enzyme's ability for hydrolysing various toxic agents (for review see Lockridge, 2015). However, with aging it can lead to neurodegeneration and is considered as an indicator of Alzheimer's disease in humans (Greig et al., 2002). Changes in AChE and BChE activities after prenatal hypoxia are also observed in blood plasma of rats at various stages of postnatal development, which might affect their immune and stress responses (Kozlova et al., 2018).

Prenatal hypoxia on E14 also affected the levels of brain expression and activity of such peptidases as neprilysin and endothelin-converting enzymes (Nalivaeva et al., 2004, 2012) and altered the adenylate cyclase system (Zhuravin et al., 2002). In particular, the enzyme activity of adenylate cyclase in the striatum, which reversely correlates with the ability of rats to learn instrumental reflexes, was much higher in rats subjected to prenatal hypoxia and correlated with their learning deficits.

Hypoxia, and prenatal hypoxia in particular, are known to regulate expression of APP whose gene has a hypoxia-responsive element (Lahiri et al., 2003). This protein plays an important role in development of the nervous system (Young-Pearse et al., 2007) and the Aβ peptide produced from its precursor has a causative role in development of Alzheimer's disease (Hardy and Selkoe, 2002). Analysis of the content of APP in rats subjected to prenatal hypoxia also revealed significant changes in the levels of this protein in the sensorimotor cortex (Nalivaeva et al., 2003). Prenatal hypoxia not only led to an increase in the content of the membrane bound form of APP at different postnatal stages of rat development but also reduced production of its soluble forms (sAPP) which have protective neuritogenic properties (for review see Chasseigneaux and Allinquant, 2012). Moreover, the most significant changes after prenatal hypoxia on E14 were observed on P10-P30 when formation of rat brain neuronal networks is the most active and any deficit of neuritogenic factors might underlie cognitive dysfunctions. These data also indirectly testify that prenatal hypoxia might modify the activity of α-secretase enzymes, which are important for releasing sAPPα and hence preventing formation of Aβ. The deficit of α-secretase after prenatal hypoxia might also explain the decreased production of soluble AChE since this activity can also be involved in AChE secretion (Nalivaeva and Turner, 1999). Moreover, maternal hypoxia in rats was shown to result in an increase in the activity of matrix metallopeptidases (MMPs) and decreases in the expression of tissue inhibitor of metalloproteinases (TIMPs) in the brain of neonatal rats, which can also underlie remodeling of neuronal circuits during brain development (Tong et al., 2010).

Although not studied in the models of prenatal hypoxia there is evidence that hypoxic conditions can alter expression of the γ-secretase complex (Liu et al., 2016) which not only regulates animal development via Notch signaling but also is a major enzyme involved in production of Aβ and Alzheimer's disease pathogenesis (Hartmann et al., 2001). Studies in transgenic mice modeling Alzheimer's disease have confirmed that prenatal hypoxia accelerates development of the pathology (Zhang et al., 2013).

One of the important factors which predisposes to formation of the sporadic form of Alzheimer's disease is the deficit of amyloid clearance (for review see Baranello et al., 2015). Our and other studies have shown that prenatal hypoxia leads to a significant deficit of the major amyloid-degrading enzyme neprilysin in rat brain at various stages of postnatal development (Nalivaeva et al., 2004, 2012; Wang et al., 2014). Together with the deficits of other amyloid-degrading enzymes e.g., endothelin-converting enzyme, angiotensin-converting enzyme and insulin-degrading enzyme, which are also affected by prenatal hypoxia or ischaemia (Nalivaeva et al., 2004), reduced NEP activity can lead to permanent insufficiency of amyloid clearance over the years and hence predispose to development of Alzheimer's disease pathology in later life (Nalivaeva et al., 2008; Wang et al., 2010).

On the other hand we observed an increased level of TTR expression in the choroid plexus of rat pups subjected to prenatal hypoxia (Vasilev et al., 2018). TTR is suggested to play a contributory role in regulation of the levels of brain Aβ (Li and Buxbaum, 2011; Du et al., 2012). Since APP expression in rat brain is also increased after prenatal hypoxia (Nalivaeva et al., 2004) it is possible that TTR increase might also function as a measure to protect the brain from potential accumulation of neurotoxic levels of Aβ and partially compensate for any reduction in NEP activity. However, TTR itself might undergo protein misfolding and aggregation leading to TTR amyloidosis (Coles and Young, 2012).

## Treatment of the consequences of prenatal hypoxia

There are several strategies to protect the developing brain against pathological effects of prenatal hypoxia which can be applied both during the pregnancy and in newborns. Some pharmacological approaches include maternal treatment with glutamate antagonists to prevent neuronal cell death caused by hypoxia-induced excitotoxicity. Epidemiological studies suggest that administration of magnesium sulfate (MgSO_4_) to pregnant women with pre-eclampsia or during pre-term labor was protective against development of cerebral palsy in infants (Schendel et al., 1996). Further animal experiments revealed that injection of pregnant mice on gestational day 17 with MgSO_4_ during 4 h prior to chamber hypoxia (9% O_2_, 2 h) reduced motor disabilities and protected cerebellar cells in the postnatal development of the offspring (Golan et al., 2004). However, this treatment also had some side effects on the cells in the cerebral cortex and hippocampus which diminishes enthusiasm toward this rather common therapeutic approach. At the molecular level in the guinea pig model of prenatal hypoxia it was shown that post-hypoxic administration of MgSO_4_ prevents increased nuclear Ca^2+^ influx and protects nuclear membrane function in neuronal cells (Maulik et al., 2005).

To prevent excessive calcium influx to neuronal cells caused by hypoxia several antagonists to voltage-sensitive calcium channels have been developed over the years. They subsequently demonstrated neuroprotective effects in the developing brain when given to pregnant females before or during hypoxic episodes (for review see Rees et al., 1998). In an experimental model of prenatal hypoxia it was shown that nimodipine was able to prevent hypoxia-induced inhibition of brain growth and long-term behavioral deficits in rat offspring (Nyakas et al., 1994) whereas flunarizine (an L-type VSCC blocker) reduced neuronal death in a model of cerebral ischaemia in neonatal rats (Gunn et al., 1989, 1994). Although flunarizine is more efficient that nimodipine in improvement of cerebral blood flow, it can cause fetal hypotension resulting in pre-term death, which diminishes its therapeutic value in pregnancy (Gunn et al., 1989). More recent clinical studies have revealed that MgSO_4_ is more effective than nimodipine in women with severe pre-eclampsia in preventing seizures while demonstrating no significant difference to the neonatal outcome (Belfort et al., 2003).

Treatment of pregnant women with synthetic glucocorticoids is a widely accepted practice to reduce systemic morbidity and mortality in infants after premature birth caused by pathologic pregnancies including prenatal hypoxic and ischaemic insults (Barrett et al., 2007; Bennet et al., 2012). Clinical studies suggest that betamethasone and dexamethasone treatment reduce the rate of neonatal morbidity and mortality. Dexamethasone was also more effective in preventing brain hemorrhage in pre-term neonates (Elimian et al., 2007). However, the impact of glucocorticoid treatment on the developing brain during pregnancy is still not sufficiently investigated and their postnatal application often results in impaired development of brain function (French et al., 2004). Existing animal studies suggest that dexamethasone treatment during pregnancy reduces brain tolerance to hypoxia (Carlos et al., 1991). Moreover, maternal hypoxia can lead to a significant decrease in the levels of expression of glucocorticoid receptors and abolishes the neuroprotective effect of dexamethasone in rat pup brain (Gonzalez-Rodriguez et al., 2014).

During pregnancy the levels of neuroactive steroids increases both in the maternal circulation and developing brain. These steroids contribute to a great extent to brain development and, under hypoxic conditions, their production is significantly increased (Hirst et al., 2009). Elevated levels of allopregnanolone, the most potent GABA_A_ receptor modulator, plays an important role in protecting the brain against excitotoxicity caused by traumatic injuries and hypoxia (Djebaili et al., 2005). Treatment of pregnant rats with allopregnanolone was shown to protect the developing fetal hippocampus against hypoxic insults (Fleiss et al., 2012) although safe protocols for its clinical applications still require refinement.

A rather promising pharmacological strategy against the damaging effects of intrauterine hypoxia and prenatal asphyxia around birth is administration of selective nNOS inhibitors which was shown to reduce cerebral palsy outcome in a rabbit model (Yu et al., 2011). The underlying mechanism of this therapeutic approach is that nNOS (but no iNOS) inhibition preserves the integrity and functioning of brain mitochondria increasing neuronal cell survival (Rao et al., 2011). This promising approach is still being tested in larger animal models (Drury et al., 2013, 2014). Recently, studies have suggested that postnatal erythropoietin treatment might also be a beneficial therapeutic strategy since its administration results in recovery of structural integrity and myelination in developing rat brain and improves behavioral and memory deficits caused by perinatal brain injury (Robinson et al., 2018).

There are some other reports on beneficial approaches which include intranasal administration to rats on postnatal days 13–15 of a homeostasis protective peptide, Pro-Gly-Pro capable of preventing the negative effects of acute prenatal hypoxia (Graf et al., 2006). The effect of this peptide was similar to the effects of other neuroprotective peptides such as semax and β-casomorphin-7 developed by scientists from Moscow State University (Maslova et al., 2001). The authors explain the observed protective effect of Pro-Gly-Pro by its ability to normalize blood supply to organs and tissues (Badmaeva et al., 2006).

Since prenatal hypoxia induces a wide range of oxidative stress reactions in developing fetal brain (Sab et al., 2013) treatment with antioxidants during hypoxic pregnancies is also beneficial against fetal metabolic impairment (Okatani et al., 2000). Given prenatally, antioxidants were shown to protect rat brain neurones against high-altitude hypoxia even in postnatal life (Wu et al., 2011). Prenatal supplementation with docosahexaenoic acid has also been found protective against neuronal damage in a rat model of perinatal hypoxia-ischaemia (Berman et al., 2009). Administration of L-arginine was also found neuroprotective for the fetal brain against maternal stress during pregnancy (Mahmoudi et al., 2016).

With regard to the early postnatal interventions against neuroinflammation caused by perinatal hypoxia, application of hypothermia and N-acetylcysteine is currently considered to be effective although the underlying neuroprotective mechanisms are not yet fully investigated (Jenkins et al., 2009). Hypothermia combined with cannabidiol treatment were also shown to provide additive protecting effect against hypoxia on neuronal metabolism in the developing brain of new-born piglets when applied shortly after the insult (Lafuente et al., 2016).

For minimizing the risk factor of insufficient oxygen supply to the fetal brain during pathological pregnancy maternal voluntary exercise was shown to be beneficial (Akhavan et al., 2012) providing long-lasting protection against neurodegeneration and AD-related pathology in later life (Herring et al., 2012).

In recent years hypoxic pre- and post-conditioning has been suggested as a non- invasive method to improve brain function after severe hypoxic episodes (for review see Rybnikova and Samoilov, 2015). This approach was also shown to be protective against the damaging effects of prenatal hypoxia (Nalivaeva et al., 2004; Basovich, 2013). The neuroprotective effect of hypoxic preconditioning was also confirmed in a chick embryo model of prenatal hypoxia via induction of HIF-1 mechanisms (Giusti and Fiszer de Plazas, 2012).

There are reports suggesting that ozone therapy, which is beneficial for treating various medical conditions e.g., peritonitis, chronic skin ulcers, infected wounds, initial gangrene and burns (Valacchi et al., 2005; Safwat et al., 2017) might have application for treatment of neurological disorders (Re et al., 2008; Frosini et al., 2012). Although ozone, as a very reactive air pollutant, can cause various complications, its safe and well-defined clinical administration might reinforce the antioxidant system of the organism and protect cells and tissues against oxidative stress (Smith et al., 2017). This is of particular importance in new-born organisms with an undeveloped endogenous antioxidant system (Davis and Auten, 2010). In relation to perinatal injuries, it was shown that ozone therapy decreases neuronal apoptosis and improves memory in rat pups subjected to hypoxic/ischaemic brain injury on P7 (Resitoglu et al., 2018). However, protective application of this approach for treatment of neurological disorders caused by prenatal hypoxia is still rather controversial.

Another complementary approach to restore the impaired brain functions caused by hypoxia in pregnancy and at birth includes pre- and postnatal environmental enrichment (Durán-Carabali et al., 2017). Enriched environment was shown to increase enzyme activity of the important neuropeptidase neprilysin capable of degrading Aβ and reducing amyloid burden in Alzheimer's disease mice as well as to upregulate expression of genes associated with learning and memory (Lazarov et al., 2005).

Searching for strategies to increase expression and activity of the amyloid-degrading enzymes, in particular of neprilysin, in rat brain we have found that valproic acid not only upregulates expression of NEP levels in the cortex and hippocampus reduced by prenatal hypoxia but also improves cognitive functions of the adult animals (Nalivaeva et al., 2012). This process involves regulation of the *NEP* gene via binding of the C-terminal fragment of APP (termed AICD) to the *NEP* promoter (Belyaev et al., 2009). In a cell model of hypoxia the reduced NEP expression and activity were shown to be related to an increased cleavage of AICD due to upregulation of caspases and this process was reversed by caspase inhibition (Kerridge et al., 2015). Increased caspase expression and activity observed in the cortex of rats subjected to prenatal hypoxia also correlated with decreased AICD and NEP levels which were restored after intraventricular administration to rats of the caspase-3 inhibitors, Z-DEVD-FMK or Ac-DEVD-CHO (Kozlova et al., 2015; Vasilev et al., 2016a). Administration of the inhibitors also resulted in prolonged improvement of learning and short-term memory in rats subjected to prenatal hypoxia up to the levels of control animals when tested in the two-level maze even one and half months after injections (Vasilev et al., 2016a).

Antioxidants, in particular epigallocatechin gallate (EGCG), which was shown to increase NEP expression in cell models (Melzig and Janka, 2003), were also found capable to improve neurological deficits in our model of rat prenatal hypoxia (Zhuravin et al., 2018) and in a mouse model of Alzheimer's disease (Chang et al., 2015). The increase in NEP activity correlated with an improvement of rat performance in the radial maze and with restoration of both short- and long-term memory in the novel object recognition test (Zhuravin et al., 2011, 2018; Nalivaeva et al., 2012).

Undoubtedly, there are other pharmacological and epigenetic approaches to restore expression of genes down-regulated by hypoxia. Using cell models we have tested several compounds (e.g., Gleevec and bexarotene) which are able to upregulate expression of neprilysin and other amyloid-clearing proteins, including a transport protein transthyretin and insulin-degrading enzyme (Kerridge et al., 2014; Nalivaeva et al., 2016). These compounds are currently being tested in our rat model of prenatal hypoxia.

## Concluding remarks

Despite intensive studies of the molecular mechanisms underlying impaired brain development and functioning caused by prenatal hypoxia we are still far from a complete appreciation of all the changes at the molecular and epigenetic levels which shape individual development in postnatal life. Further studies using models of prenatal hypoxia will allow us and others to gain a deeper insight into the mechanisms of dysregulation of neuronal functions during fetal development and design new preventive strategies to restore brain integrity and cognitive functions.

## Author contributions

NN has outlined and written the manuscript, AT has discussed and edited the manuscript, IZ has selected literature and discussed the manuscript.

### Conflict of interest statement

The authors declare that the research was conducted in the absence of any commercial or financial relationships that could be construed as a potential conflict of interest.
